# Local Tuberculous Abscess in a Bull

**Published:** 1892-11

**Authors:** Wyatt Johnston

**Affiliations:** Montreal


### LOCAL TUBERCULOUS ABSCESS IN A BULL.*

By Wyatt Johnston, M.D., Montreal.

The importance which now attaches to the early recognition of tuberculosis in herds of valuable cattle makes it of interest to record the following unusual case.

On March 11th, 1890, I received from Dr. McEachran, of Montreal, a specimen of pus with the statement that it came from a small abscess about the jaw of a valuable thoroughbred bull, and the owner of the animal feared that it might be suffering from tu-

* Paper presented at the 29th Annual Meeting of the United States Veterinary Medical Association.

berculosis. The history of the stock did not give any evidence of tuberculosis having occurred among the other animals, though it had appeared about two years before on an adjoining farm, and the present subject had served a cow in this herd during the previous season. The present animal, a fine, strong, healthy-looking bull, four years old, was always in perfect health till two months previous, when the attendant noticed a swelling beneath the jaw. This suppurated and was opened, but did not heal up. Recently another small abscess formed in the neck, which also did not heal when opened. The attendant thought that the inflammation was caused by the chafing of a rope around the animal's neck in the stall.

The pus when received was grayish and oily-looking, like freshly mixed paint, with faint streaks of red. It contained numerous bright yellow particles the size of pin heads, and some small gritty bodies of about the same size. Microscopically, it was composed of leucocytes, a few red blood cells and fat crystals. The yellow particles proved not to be actinomyces. Cover glass preparations (10 in number) were examined without detecting tubercular bacilli. Some of the covers were left as long as eighteen hours in the stain. No other bacteria appeared to be present.

I inoculated three rabbits in the anterior chamber and three guinea pigs in the peritoneal cavity, using one to two drops in each case. A guinea pig and a rabbit were placed for control in the cages with the inoculated animals.

On March 15th I visited the farm in company with the owner and Prof. McEachran. The bull was found to be a fine, healthy-looking, well-nourished animal. Beneath the jaw were two small suppurating abscess cavities on the left side, with red firm granulating walls. Lymph glands in the neck not enlarged. Physical examination showed no evidences whatever of tuberculosis. In spite of Dr. McEachran's recommendation to delay a few weeks till we learned the result of the inoculation, keeping the bull meanwhile under observation and carefully isolating him, the owner insisted on his being slaughtered immediately, together with a yearling bull, the son of the one under consideration. Accordingly we had an opportunity of making an autopsy on both animals forthwith, but failed to detect any evidences of tuberculosis in either the internal organs or glands. The penis, testes and vasa deferentia were free from tubercles. The abscess cavities appeared to be purely subcutaneous in location and showed no evidences of tubercles or caseous masses in their vicinity. The bodies of the vertebræ were free from tubercles. As the meat was intended for sale, the muscles and intermuscular planes were only examined as far as could be done while the carcass was being dressed.

My notes of the inoculation are as follows:

On March 16th, one of the rabbits shows panophthalmitis. In the other two the corneal wound is nearly healed, but slight plastic iritis persisted.

March 18th. Examined sections made through the portions of the abscess wall after hardening in alcohol. These showed a superficial necrotic zone surrounded by granulation tissue formed of small round cells. No giant cells. A large number of sections were examined for tubercular bacilli without finding any. (This examination was again repeated on April 24th, still with a negative result. The absence of actinomyces was also confirmed.)

April 11th. One of the guinea pigs has been wheezing for a week and losing flesh rapidly. Died to-day. Autopsy. Over parietal peritoneum a few pearly-gray nodules. Omentum shows small gray granulations with opaque centres. Spleen contains yellow-white opaque nodules about the size of split peas. Microscopic examinations of sections of the spleen shows a few tubercle bacilli in every field.

April 24th. All the animals killed to-day. The control animals found to be free from tuberculosis. The two remaining guinea pigs present similar conditions to the one which died on April 11th, tubercle bacilli being found in the spleen in each case.

The three rabbits all showed extensive tuberculous inflammation in the regions of the iris. Tubercular bacilli were found in each case. Two of the rabbits showed caseous nodules in the lungs and spleen which contained numerous tubercle bacilli.

I have reported this case in detail because I have not been able to hear of a similar one when the disease was limited in this peculiar manner.

It is of interest, owing to the conclusive results of the inoculation experiments when a most careful microscopical examination had failed to detect tubercle bacilli. The apparently purely local nature of the lesions is also interesting. It is seldom that an opportunity of examining a valuable animal post mortem is insisted on by the owner in such an obscure case. From the appearance of the abscess wall, it is probable that recovery would have been complete had the animal been allowed to live. Unfortunately the suspicions of the owner were well grounded, and he lost in the following year a valuable cow from tuberculosis. In this case a large fungating, ulcerating growth, as large as an egg, projected from the vocal cords, and Dr. J. B. Paige, of Amherst, who examined the organs, was able to demonstrate the masses of tubercle bacilli actually in pure culture in necrotic spots from the larynx and liver.

It would have been preferable had I also made inoculations with material from the abscesses, taken at the time of the autopsy under aseptic precautions. The fact, however, that of the material sent me six separate samples each gave positive inoculation results, seems to leave no doubt that the infection was in no way due to extraneous contamination. It must be remembered that at the time of the autopsy we came to the conclusion that the case was not one of tuberculosis, and therefore no further information was sought.

The fact that two separate cases of tuberculosis in unusual localities in this herd occurred within a year, seems to indicate some unusual source of infection. None of the farm hands were tuberculous, as far as could be learned. Possibly the infection may have been accomplished through the agency of some smaller animals, rodents for instance, about the farm, in whom tuberculosis would not be suspected. The fact that no subsequent cases appeared in the herd this year, points to some transient source of infection of an unusual character.

